# Evolution of transcriptional control of antigenic variation and virulence in human and ape malaria parasites

**DOI:** 10.1186/s12862-021-01872-z

**Published:** 2021-07-08

**Authors:** Mackensie R. Gross, Rosie Hsu, Kirk W. Deitsch

**Affiliations:** Department of Microbiology and Immunology, Weill Cornell Medical College, New York, NY USA

**Keywords:** Cytoadherence, Transcriptional regulation, Mutually exclusive expression, Pathogenesis

## Abstract

**Background:**

The most severe form of human malaria is caused by the protozoan parasite *Plasmodium falciparum*. This unicellular organism is a member of a subgenus of Plasmodium called the *Laverania* that infects apes, with *P. falciparum* being the only member that infects humans. The exceptional virulence of this species to humans can be largely attributed to a family of variant surface antigens placed by the parasites onto the surface of infected red blood cells that mediate adherence to the vascular endothelium. These proteins are encoded by a large, multicopy gene family called *var*, with each *var* gene encoding a different form of the protein. By changing which *var* gene is expressed, parasites avoid immune recognition, a process called antigenic variation that underlies the chronic nature of malaria infections.

**Results:**

Here we show that the common ancestor of the branch of the *Laverania* lineage that includes the human parasite underwent a remarkable change in the organization and structure of elements linked to the complex transcriptional regulation displayed by the *var* gene family. Unlike the other members of the *Laverania*, the clade that gave rise to *P. falciparum* evolved distinct subsets of *var* genes distinguishable by different upstream transcriptional regulatory regions that have been associated with different expression profiles and virulence properties. In addition, two uniquely conserved *var* genes that have been proposed to play a role in coordinating transcriptional switching similarly arose uniquely within this clade. We hypothesize that these changes originated at a time of dramatic climatic change on the African continent that is predicted to have led to significant changes in transmission dynamics, thus selecting for patterns of antigenic variation that enabled lengthier, more chronic infections.

**Conclusions:**

These observations suggest that changes in transmission dynamics selected for significant alterations in the transcriptional regulatory mechanisms that mediate antigenic variation in the parasite lineage that includes *P. falciparum*. These changes likely underlie the chronic nature of these infections as well as their exceptional virulence.

**Supplementary Information:**

The online version contains supplementary material available at 10.1186/s12862-021-01872-z.

## Background

Eukaryotic parasites of the genus *Plasmodium* infect a broad range of vertebrate species, including birds, reptiles and mammals [1]. There are five species that infect humans and cause malaria, a disease that can be severe, resulting in significant morbidity and mortality within human populations in tropical and subtropical regions of the world [2]. Malaria parasites exhibit multiple morphological states as they transition between their mosquito vectors and their vertebrate hosts, but all symptoms of the disease result from asexual replication of the parasites within the circulating red blood cells (RBCs) of the infected individual. This stage of the infection is associated with a massive increase in parasite numbers and can lead to severe anemia, extensive inflammation and vascular occlusion that can disrupt circulation [3]. The influence of malaria on human evolution is thought to have been substantial, and infection by Plasmodium parasites has been proposed to have played a significant role in shaping the human genome [4].

Of the five parasite species that infect humans, by far the most virulent is *P. falciparum*. The virulence of this species is due in large part to the cytoadhesive properties of infected RBCs that result from the placement on the RBC surface of parasite encoded variable antigens, included proteins referred to as RIFIN (repetitive interspersed family), STEVOR (subtelomeric variant open reading frame), PfMC-2TM (*P. falciparum* Maurer’s cleft-2 transmembrane domain proteins) and PfEMP1 (*P. falciparum* erythrocyte membrane protein 1) [5, 6]. The contribution of RIFINs, STEVORs and PfMC-2TMs to cytoadherence is unclear, however PfEMP1 has been shown to be the major cytoadhesive molecule and is thought to be the primary virulence determinant of *P. falciparum* [7]. *P. falciparum* is the sole human-infective member of a subgenus of *Plasmodium* that infects apes called the *Laverania*. Unlike malaria species from other evolutionary lineages, the seven characterized species of the *Laverania* are unique for their expression of EMP1 on the surface of infected RBCs [7] where it binds to molecules on the host endothelial surface and enables the infected cell to adhere to blood vessel walls, thus sequestering the parasites from the peripheral circulation and avoiding destruction in the spleen [8]. However, placement of EMP1 on the RBC surface stimulates the host to generate antibodies against this protein, thus parasites must continuously vary the form of EMP1 they express to avoid clearance by the adaptive immune response. This phenomenon is referred to as antigenic variation, and it is remarkably effective at enabling parasites to maintain chronic infections that can last over a year [3, 9–11].

The ability of the *Laverania* parasites to continuously change the expressed form of EMP1 over the course of an infection is rooted in the expansion of the genes encoding different forms of the protein into large, multicopy gene families called *var*. A recent comparative study of the genomes of seven *Laverania* species identified *var* gene families ranging in size from 28 to 105 copies, with the genes organized into clusters found within the subtelomeric regions near the chromosome ends or in tandem arrays within the internal regions of the chromosomes [12]. Extensive studies in *P. falciparum* indicate that the genes are transcribed in a mutually exclusion fashion, with all but a single gene maintained in condensed heterochromatin and thus transcriptionally silent [13–15]. By switching the single *var* gene that is actively transcribed, parasites change the form of EMP1 expressed on the infected cell surface and thereby undergo antigenic variation. Whole genome analysis of many *P. falciparum* isolates also indicates that the *var* gene family is highly recombinogenic [16, 17], resulting in rapid diversification of the EMP1 coding regions and thus preventing the acquisition of cross-reactive immunity in geographical regions with high transmission rates. The *var* gene family therefore represents a key attribute of the *Laverania* that has substantially contributed to its evolutionary success and the increased virulence displayed by *P. falciparum* compared to other human-infective species of malaria parasites.

The *var* gene family is present in the genomes of all seven known members of the *Laverania* subgenus, indicating that it evolved in the common ancestor of these parasites. Interestingly however, in their recent comparative study, Otto and colleagues documented a remarkable change in the organization of protein domain structures within the EMP1 proteins encoded by different clades within the *Laverania* subgenus [12]. Specifically, they found that four species that constitute a clade which includes *P. falciparum* have diverged significantly in EMP1 protein structure, suggesting that parasites from this clade cytoadhere to different host endothelial receptors when compared to the more distantly related members of the subgenus. If correct, this marks a significant moment in the evolution of this lineage of pathogens, particularly since this alteration in cytoadhesive properties directly shaped the virulence of the human parasite, *P. falciparum*. The selective forces that led to this change in EMP1 structure are unknown, however Otto et al. found that an unusual and highly conserved *var* gene called *var2csa* appears to be the sole remnant of the more ancient type of EMP1 remaining in *P. falciparum*. This gene was originally identified as encoding a form of EMP1 that binds to receptors in the placenta [18], but more recently has also been proposed to function as an intermediate in *var* transcriptional switching [19, 20]. They therefore hypothesized that this ancient gene has been maintained as a single copy in *P. falciparum* to serve the dual functions of cytoaherence within the placenta as well as regulating *var* gene transcriptional switching [12]. This study highlighted key steps in the evolution of the *var* gene family, both in terms of the virulence linked to cytoadherence by EMP1 and the transcriptional regulation that enables antigenic variation.

Here we extend these findings to investigate the evolution of other elements implicated in transcriptional regulation of the *var* gene family. We found that both the regulatory proteins previously implicated in the control of *var* gene transcription and a unique regulatory element found within the conserved intron of *var* genes are present throughout the entire *Laverania* subgenus, indicating that many aspects that control *var* gene expression evolved in the common ancestor of this entire group of parasites. In contrast, the divergence of *var* upstream regulatory regions into distinct subtypes linked to virulence, as well as the evolution of *var2csa* and a second strain-transcendent *var* gene called *var1csa*, occurred relatively recently within the *Laverania* subgenus, within the clade that includes *P. falciparum*. These changes all appear to be related to the evolution of a more structured transcriptional regulatory mechanism, potentially enabling parasites to more precisely coordinate the process of antigenic variation. Phylogenetic analysis indicates that this change in the structure of the *var* gene family coincides with a well-documented alteration in African climatic conditions that likely altered parasite transmission dynamics. Thus, the selective pressures that shaped the evolution of virulence of the sole human-infective member of the *Laverania* lineage can be traced to two key events, one involving a change in host receptor recognition for cytoadherence and a second that influenced how antigenic variation is controlled.

## Results

### Proteins implicated in *var* gene regulation are found encoded in the genomes of all *Laverania* species

Given the importance of *var* genes and EMP1 to the virulence of *P. falciparum*, and the recent identification of significant changes in EMP1 structure over the course of the evolution of the *Laverania* [12], we were interested in systematically considering the evolution of the mechanisms controlling *var* gene regulation and antigenic variation in the parasite lineage that gave rise to *P. falciparum*. While the precise mechanisms that control *var* gene activation, silencing and mutually exclusive expression are not completely understood, numerous proteins have been implicated in various aspects of *var* gene regulation, included the histone methyltransferases SET2 [21, 22] and SET10 [23], the demethylase JmJC1 [22, 24], the histone deacetylases SIR2A and SIR2B [25–27], the translation factor PTEF [28] and the RECQ helicases RQ1 and WRN [29, 30]. Syntenic orthologues of each protein are found throughout the *Laverania*, and with the exception of PTEF, all can also be identified in the non-*Laverania* parasites *P. vivax, P. berghei* and *P. gallinaceum* (Table 1), therefore they must have existed in the common ancestor of all Plasmodium species. These proteins represent orthologues of factors known from model organisms to play roles in chromatin assembly, transcriptional regulation or aspects of DNA repair, thus they are likely to have broader roles in parasite nuclear functions beyond their functions in *var* gene regulation. The histone methyltransferase SET2 and its cognate demethylase JmJC1 are similarly found throughout the *Laverania* as well as in *P. vivax* and the bird parasite *P. gallinaceum*, however they are not encoded in the rodent parasite genomes (Table 1). It was previously suggested that these proteins were specific to primate parasites [22], however the availability of whole genome sequences from additional parasite species has identified syntenic orthologues in parasites of birds, suggesting an origin prior to the root of the Plasmodium genus and more general functions not specific for *var* gene regulation.

**Table 1:** Orthologous genes encoding proteins implicated in *var* gene regulation

	SET10	Sir2A	Sir2B	WRN	RQ1	SET2	JmJC1	PTEF
*P. alderi*	PADL01_1221600	PADL01_1327800	PADL01_1451400	PADL01_1429900	PADL01_0917600	PADL01_1321000	PADL01_0809000	PADL01_0201200
*P. gaboni*	PGABG01_1219800	PGABG01_1327000	PGABG01_1450200	PGABG01_1428600	PGABG01_0916000	PGABG01_1320200	PGABG01_0809800	PGABG01_0200100
*P. blacklocki*	PBLACG01_1220400	PBLACG01_1327100	PBLACG01_1450800	PBLACG01_1429300	PBLACG01_0918200	PBLACG01_1320300	PBLACG01_0809600	*
*P. billcollinsi*	PBILCG01_1221100	PBILCG01_1329300	PBILCG01_1450900	PBILCG01_1430500	PBILCG01_0922000	PBILCG01_1322500	PBILCG01_0815300	PBILCG01_0205100
*P. reichenowi*	PRG01_1224300	PRG01_1331000	PRG01_1451400	PRG01_1429900	PRG01_0926000	PRG01_1324200	PRG01_0812700	PRG01_0201700
*P. praefalciparum*	PPRFG01_1233800	PPRFG01_1330800	PPRFG01_1452100	PPRFG01_1430600	PPRFG01_0917400	PPRFG01_1324000	PPRFG01_0811300	PPRFG01_0202500
*P. falciparum*	PF3D7_1221000	PF3D7_1328800	PF3D7_1451400	PF3D7_1429900	PF3D7_0918600	PF3D7_1322100	PF3D7_0809900	PF3D7_0202400
*P. vivax*	PVX_123685	PVX_082385	PVX_117910	PVX_085045	PVX_099345	PVX_116765	PVX_123283	*missing*
*P. berghei*	PBANKA_1436200	PBANKA_1343800	PBANKA_1315100	PBANKA_1014800	PBANKA_0819500	*missing*	*missing*	*missing*
*P. gallinaceum*	PGAL8A_00535100	PGAL8A_00241100	PGAL8A_00210600	PGAL8A_00301500	PGAL8A_00431400	PGAL8A_00234100	PGAL8A_00526200	*missing*

The species names are listed on the left and the gene annotation number for each protein are listed in columns under each protein name. Genes encoded SET2 and JmJC1 are not found at the syntenic genomic positions in *P. berghei*, and syntenic genes encoding PTEF are not found in either *P. vivax, P. berghei* or *P. gallinaceum* (denoted with italicized text). *The existence of PTEF in *P. blacklocki* could not be determined because the syntenic region of the genome is incomplete in the most recent genome sequence assembly for this species

In contrast to the other regulatory proteins associated with *var* gene regulation, PTEF (Plasmodium translation enhancing factor) appears to be specific to the *Laverania* (Table 1). BLAST searches of the genomes of non-*Laverania* parasite species failed to identify genes with significant similarity, and the syntenic genomic positions of *P. vivax, P. berghei* and *P. gallinaceum* do not contain open reading frames encoding similar proteins. Thus, the evolution of PTEF appears to be coincident with the appearance of *var* genes in this branch of the *Plasmodium* genus. In *P. falciparum*, PTEF has been described as a protein specifically upregulated during pregnancy where it enhances translation of the EMP1 encoded by the unique *var* gene called *var2csa* [28]. *var2csa* is unusual in that the gene includes a regulatory upstream open reading frame (uORF) that can suppress translation of the EMP1 encoding exons [31, 32]. PTEF was shown to interact with the translating ribosome complex to enable translational reinitiation [28], thereby overcoming the translational repression of the uORF and promoting expression of a form of EMP1 that binds to the proteoglycan chrondroitin sulfate A on the syncytiotrophoblasts of the placenta [33]. *var2csa* was therefore proposed to be translationally repressed in the absence of a placenta through the uORF, but expression of PTEF when parasites infect pregnant women relieved this repression and enabled parasites to take advantage of this new anatomical site for cytoadhesion. However, *var2csa* and its regulatory uORF appear to have evolved relatively late in the *Laverania* (see below), while PTEF is present in all members of the subgenus, indicating that PTEF must have a function in addition to or beyond regulation of *var2csa* translation. A recent genome-wide assessment of *P. falciparum* transcripts noted that the number of potential uORFs is remarkably high [34] and given the substantial degree of synteny and sequence homology within the entire subgenus, this extends to the other *Lavarania* species as well. The role of PTEF in overcoming the repressive effects of uORFs on mRNA translation therefore could include a broader class of mRNAs beyond *var2csa*, thus explaining its conservation in parasites that do not possess this particular *var* gene.

### The regulatory element found in *var* introns is conserved throughout the *Laverania* subgenus

**Fig. 1:**
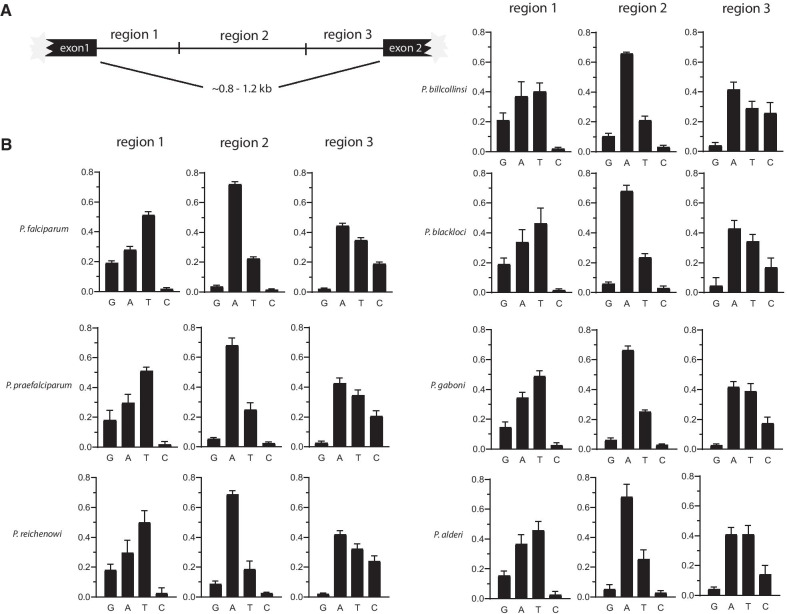
Conservation of *var* intron structure in all seven *Laverania* species. **A** Schematic showing the division of *var* introns into three regions based on base content and strand asymmetry. **B** Calculation of base content of the positive strand of *var* introns for each species of the *Laverania*. Note the prominent G vs C asymmetry in regions 1 and 3 and the conservation of this asymmetry in all seven species

Initial characterization of *var* genes in *P. falciparum* identified a conserved, two exon structure that includes an unusual intron that separates the extracellular domain of EMP1 from the intracellular portion that anchors the protein in the RBC membrane [35]. This intron has independent promoter activity, giving rise to long, noncoding RNAs of unknown function [35–37], and it has been implicated in the regulation of *var* gene silencing and mutually exclusive expression [38, 39], although the precise mechanism remains undefined [40]. Close examination of the sequence of *var* introns identified a distinct strand asymmetry, with each intron easily divided into three well defined regions based on base content, with regions 1 and 3 displaying “mirror images” of one another [41]. The strand asymmetry is easily discernible through the display of G vs C and A vs T content within the separate regions, with region 1 containing high G content and virtually no Cs and region 3 displaying the inverse relationship, with respect to the positive DNA strand (Fig. 1). A bi-directional promoter driving expression of long, noncoding RNAs has been mapped to region 2 [36, 41]. This conserved structure is found within the introns of all *var* genes in *P. falciparum*, with the exception of an unusual type called *var3* or “type 3” *var* genes. Type 3 *var* genes have been identified in some, but not all, strains of *P. falciparum*, and their function is unknown [42–44]. The introns of these genes lack region 2.

To determine if the regulatory element found in the *var* introns of *P. falciparum* is also observed in *var* genes from parasites throughout the *Laverania* subgenus, we obtained *var* intron sequences from annotated *var* genes from the 6 other *Laverania* species. Each intron sequence was divided into three regions and the average base content calculated for each region as previously described [41]. The base pair content for each region was displayed as histograms (Fig. 1) and all seven species show virtually identical patterns of strand asymmetry. The unusual base pair structure of *var* introns originally observed in *P. falciparum* therefore is also found in *var* genes throughout the *Laverania* and likely extends to the origins of *var* genes themselves. With the exception of the Type 3 *var* genes mentioned above, the intron structure does not appear to vary based on the chromosomal location or gene type, consistent with what was previously reported for *P. falciparum* [45], Interestingly, similar to a previous report [46], we were unable to identify Type 3 *vars* in species other than *P. falciparum*, although more complete genome assemblies of these species might enable identification of *var* genes that are currently missing.

### Evolution of strain-transcendent *var* genes in a subset of *Laverania* species that includes *P. falciparum*

**Fig. 2:**
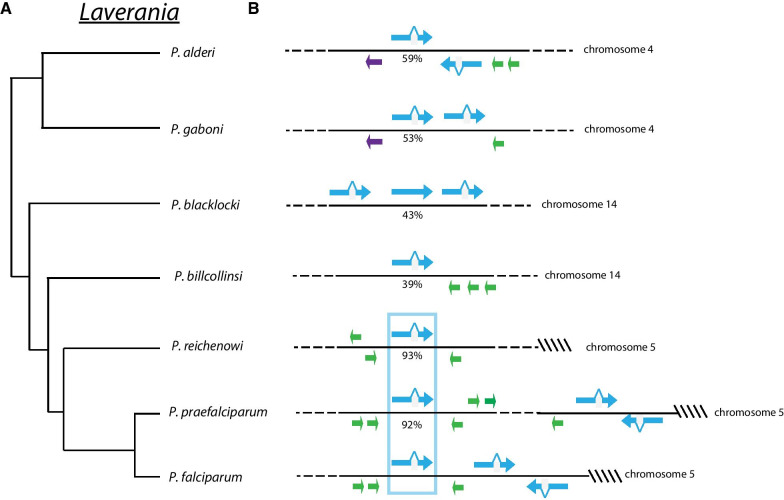
Conservation of the unusual *var* gene *var1csa* in *P. reichenowi*, *P. praefalciparum* and *P. falciparum*. **A** A phylogenetic tree of the *Laverania* as described by Otto et al. [12]. **B** Identification of *var1csa* in *P. falciparum* (isolate SN1) and its orthologues in *P. reichenowi* and *P. praefalciparum*. The chromosomal region from each species containing the *var* gene with the highest sequence identity to *var1csa* from *P. falciparum* is shown. *var* genes are shown in blue and members of other multicopy gene families are shown in green. The genes with the highest sequence identity to *var1csa* in each species are aligned, and the percentage identity is shown below the gene. For *P. reichenowi* and *P. praefalciparum*, sequence identity exceeds 90%, and the genes display clear synteny (boxed in blue). For the remainder of the *Laverania*, the sequence identity is substantially lower and the most similar gene is not located in the syntenic position, indicating these species do not contain true orthologues of *var1csa*

The *var* gene family has been extensively studied in *P. falciparum*, including the vast diversity of individual genes when the complete genome sequences of multiple, independent geographical isolates were compared [44]. Attempts to measure the level of *var* sequence diversity within populations of naturally circulating parasites have similarly described enormous variation [9, 47] that appears to result from frequent segmental recombination events between genes [48, 49], a phenomenon documented in cultured parasites [50–52]. Remarkably, two *var* genes appear to escape recombination with other members of the *var* gene family and remain highly conserved in all *P. falciparum* isolates. The first of these genes, called *var1csa*, is found near the “right” end of chromosome 5 at the boundary between the subtelomeric region and the core genome. Unlike other members of the *var* gene family, it is transcribed late in the asexual cycle and appears to not be subjected to mutually exclusive expression [53, 54]. In addition, in all isolates examined to date, the gene carries a premature stop codon in exon 2, leading to its annotation as a pseudogene, and in the 3D7 reference genome the gene is truncated due to a subtelomeric deletion event. To determine if the conservation of *var1csa* extends beyond *P. falciparum*, we examined the syntenic region of chromosome 5 in all 7 members of the *Laverania* subgenus. We found *var* genes with 92% and 93% sequence identity at this genomic position in *P. praefalciparum* and *P. reichenowi*, respectively, but no genes with similar levels of identity in any other species (Fig. 2). BLAST searches of the complete genome sequences from the remaining species to determine if *var1csa* might exist at an alternative genomic position only identified non-conserved *var* genes with substantially less sequence identity (Fig. 2), indicating that *var1csa* is limited to *P. praefalciparum*, *P. reichenowi* and *P. falciparum*. Interestingly, the gene in *P. praefalciparum* and *P. reichenowi* also contains a predicted premature stop codon in exon 2. The selective pressure maintaining this unique *var* gene is unknown but seems unlikely to be related to its protein coding capacity given the unusual transcription pattern displayed by the gene in *P. falciparum* as well as the premature stop codon.

**Fig. 3:**
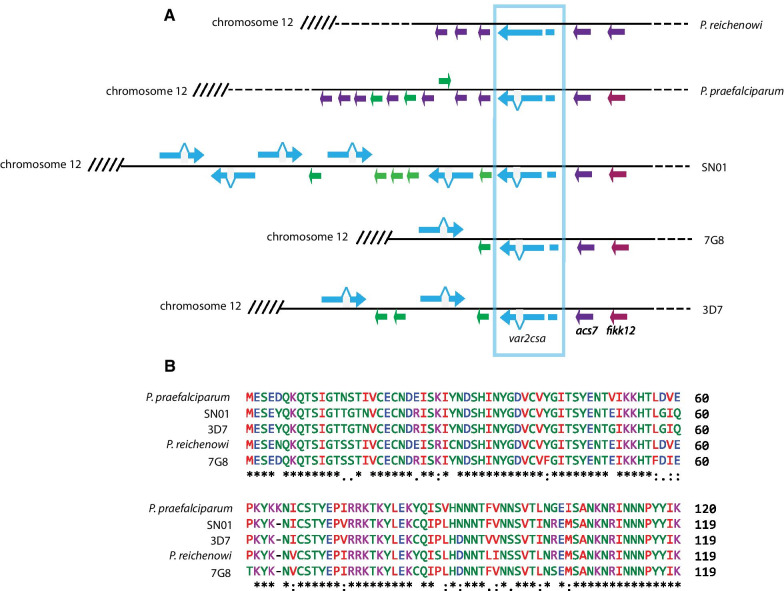
Conservation of the *var* gene *var2csa* in *P. reichenowi*, *P. praefalciparum* and *P. falciparum*. **A** Schematic showing the syntenic region of chromosome 12 in *P. reichenowi*, *P. praefalciparum* and three isolates of *P. falciparum* (SN01, 7G8 and 3D7). The positions of the single copy genes *fikk12* and *acs7* are shown and represent the border between the core genome and the highly variable subtelomeric domain. The orthologues of *var2csa* are aligned and boxed in blue. **B** Alignment of the amino acid sequence encoded by the uORF of *var2csa* from *P. praefalciparum*, *P. reichenowi* and three isolates of *P. falciparum* (SN01, 7G8 and 3D7)

Similar to *var1csa*, *var2csa* is conserved in the genomes of all *P. falciparum* isolates described to date [16]. The gene is duplicated in some isolates of *P. falciparum* and was also observed as multiple copies in the sequenced isolate of *P. praefalciparum* [44, 55], but all isolates of *P. falciparum* maintain a syntenic copy at the boundary between the subtelomeric region and the core genome at the “left” end of chromosome 12 (Fig. 3A). Also similar to *var1csa*, *var2csa* is conserved in *P. praefalciparum* and *P. reichenowi*, specifically a conserved copy is located at the syntenic position of the left end of chromosome 12, but no copies of *var2csa* are found in the other members of the *Laverania* subgenus. As mentioned above, *var2csa* is unique amongst *var* genes in its inclusion of a 360 bp uORF within the 5’ leader of its mRNA. This uORF serves as a translational repressor of the EMP1 encoded by the main open reading frame [31, 32] and is also highly conserved in all three species. This conservation extends to the amino acid sequence of the peptide encoded by the uORF (Fig. 3B). This gene was originally identified for the role of the encoded form of EMP1 in binding to the host cell surface receptor chrondroitin sulfate A in the placenta [18], however more recently it has been suggested to play a role in mediating or coordinating *var* gene expression switching, potentially as a default gene or node in a hypothetical *var* gene switching network [19, 20, 22]. In a more recent analysis of the domain structure of EMP1 proteins throughout the *Laverania*, Otto and colleagues identified a surprising change in structure of the domains that make up EMP1 in the clade that includes *P. billcollinsi*. *P. reichenowi*, *P. praefalciparum* and *P. falciparum* [12]. However, the EMP1 encoded by *var2csa* appears to be more closely related to those found in the more distantly related *Laverania* parasites, leading them to suggest that it might be the sole remnant of an ancient form of the protein that remains within the genomes of these parasites [12]. They further suggested that the selective pressure that has prevented this particular gene from recombining with other members of the family could be related to its putative role in transcriptional regulation rather than in the cytoadherence properties of the encoded protein. If correct, this suggests that the function of *var2csa* in regulating *var* gene transcription evolved in the common ancestor of *P. reichenowi*, *P. praefalciparum* and *P. falciparum*, and implicates the uORF in this function.

### Divergence of *var* gene regulatory regions into different types

With the initial completion and assembly of the *P. falciparum* reference genome [56], it became evident that *var* genes are typically found in one of three genomic positions: in subtelomeric regions transcribed away from the telomere, in subtelomeric regions transcribed toward the telomere, or in tandem arrays found within the interior of the chromosomes. The significance of this arrangement was not immediately apparent since all *var* genes, regardless of their position in the genome, appear to be subject to similar transcriptional regulation, including epigenetically regulated activation and silencing, as well as mutually exclusive expression. However, more detailed analysis of the upstream regulatory regions of all members of the family found that they could be organized into three basic types, called UpsA, B or C, based on DNA sequence similarity [45, 57], and that these types correlated closely with the genes’ position within the chromosomes. Two additional types, called UpsD and E, are associated with *var1csa* and *var2csa*, respectively. The importance of Ups type was suggested by studies of clinical samples obtained from individuals with either severe or asymptomatic disease. Parasites isolated from children with severe disease were most often found to express UpsA or B genes [58–60], while UpsC genes were not specifically associated with disease condition. Studies with cultured parasites found that UpsA and B genes also displayed higher switching rates than observed for UpsC genes, which are more stable once activated [61, 62]. These studies suggest that the upstream transcriptional regulatory regions have evolved into different types to influence how expression of the gene family progresses over the course of an infection.

Given the potential importance of the organization of the upstream regions for regulating *var* gene expression in *P. falciparum*, we investigated when this organization evolved in the *Laverania* lineage and whether a similar organization exists in other members of the subgenus. Similar to the previous analyses that identified the UpsA/B/C/D and E types [45, 57], we constructed maximum-likelihood phylogenetic trees to identify subgroups of upstream regulatory domains based on sequence similarities. Using this method, we readily detected the three primary types (UpsA/B/C) in *P. falciparum*, and similarly found them to generally correlate with genomic position, thus replicating the previously published analyses [45, 57] (Additional file 1, Fig. 1A). Applying the same methodology to the *var* gene upstream regions from *P. praefalciparum* and *P. reichenowi*, we were similarly able to detect clearly differentiated upstream types, yielding phylogenetic trees with separated branches that again loosely correlated with genomic position (Additional File 1, Fig. 1B, C) suggesting that in these species the *var* gene family is organized into Ups types similar to *P. falciparum*. For the other four *Laverania* species, fewer full-length upstream *var* sequences were available, however similar analysis yielded trees with less well defined branches and with the upstream regulatory regions from the different chromosomal locations distributed throughout the trees (Additional file 1, Fig. 1D–G), suggesting that these regulatory regions are either not organized into separate Ups types, or that the organization is less strictly defined.

**Fig. 4:**
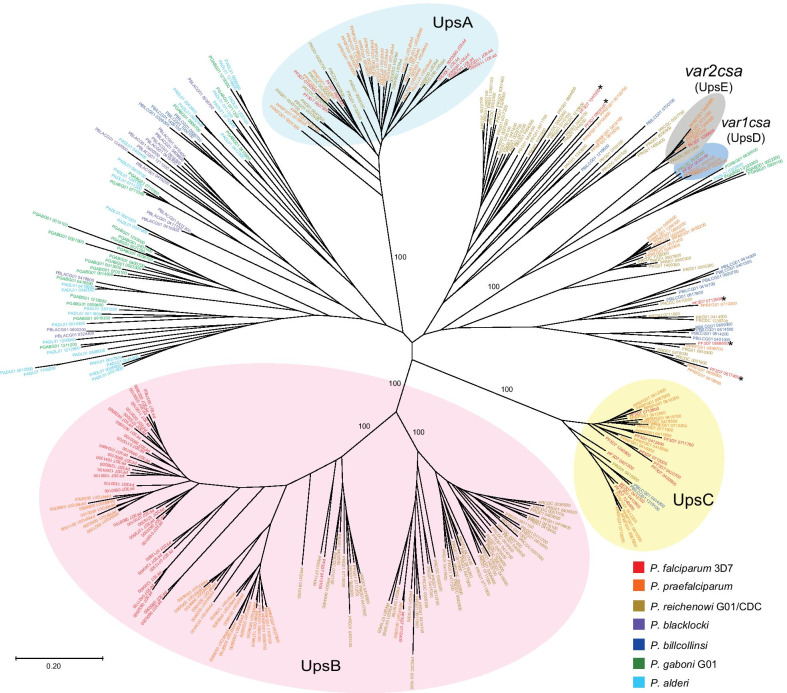
Maximum-likelihood phylogenetic tree of 0.5–1.5 kb upstream regulatory regions of *var* genes from seven *Laverania* species. The annotation number for each gene (from Plasmodb.org) is shown and the colour of the text signifies the species, as shown in the lower right panel. Sequences of the UpsB type are shaded in pink, UpsA in light blue, UpsC in yellow, *var2csa* in gray and *var1csa* in dark blue. Five genes with atypical upstream sequences from *P. falciparum* that segregate outside of the UpsA/B/C/D/E groups are marked with an asterisk. The evolutionary history was inferred using the Maximum Likelihood method and Jukes–Cantor model [63], with bootstrap values for 1000 replicates shown for various nodes. The tree is drawn to scale, with branch lengths measured in the number of substitutions per site. Evolutionary analyses were conducted in MEGA X [64]

To more closely investigate the origins of the Ups promoter type organization, we combined the *var* upstream regulatory sequences from all seven *Laverania* species and constructed a single maximum-likelihood phylogenetic tree (Fig. 4). Of particular interest are the branches that represent the UpsA and B sequences. Both of these *var* regulatory types segregate into groups that are relatively distant from the other members of the *var* gene family. More importantly, these branches only include sequences from *P. falciparum*, *P. praefalciparum* and *P. reichenowi*, with no sequences from the other four species represented within these branches. This indicates that the UpsA and B promoter types evolved at the root of the branch of the *Laverania* that includes *P. praefalciparum*, *P. reichenowi* and *P. falciparum*, coinciding with the evolution of *var1csa* and *var2csa* into distinct, strain and species transcendent genes. UpsC sequences are similarly found within a distinct branch that includes *P. falciparum*, *P. praefalciparum* and *P. reichenowi*, but this group also includes two sequences from *P. billcollinsi*, indicating that the UpsC type evolved somewhat earlier than either UpsA or B.

Interestingly, while the highly conserved aspects of *var2csa*, including the presence of the uORF in the 5’ UTR of the transcript, its location in the subtelomeric region of chromosome 12 and the structure of the encoded form of EMP1, all originated in the common ancestor of *P. falciparum*, *P. praefalciparum* and *P. reichenowi* (see Fig. 3), the upstream region of *var2csa*, also called UpsE, invariable clusters with *var* genes from the more distant *Laverania* species (Fig. 4). This indicates that *var2csa* evolved from a type of *var* gene that is no longer found in *P. falciparum,* thus explaining why it departs from the UpsA/B/C organization. This is consistent with the conclusion of Otto and colleagues, based on the structure of the encoded protein, that *var2csa* is the sole remaining example in *P. falciparum* of an ancient *var* gene [12]. We similarly found that the upstream region of *var1csa* (called UpsD) also segregates with genes from the more distant *Laverania* species, suggesting that it too might be derived from an ancient *var* gene type that is no longer found in *P. falciparum*. In addition, the Ups regions from five *P. falciparum var* genes (Pf3D7_0809100, Pf3D7_1240300, Pf3D7_0617400, Pf3D7_0712900, Pf3D7_080600) segregate within branches apart from the UpsA/B/C organization that includes the rest of the *P. falciparum var* gene family. Four of these Ups regions were previously noted by Lavstsen et al. to have atypical sequences [45], and they are all located at the boundaries between *var* gene containing chromosomal segments and the core genome, within the interior regions of the chromosomes. Genes at such positions are likely to be more constrained in their ability to undergo recombination with other family members due to the adjacent unique, highly conserved sequences, thus preventing them from diverging as rapidly. This is consistent with the clustering of these Ups regions with sequences from the more distantly related *P. reichenowi* and *P. praefalciparum* rather than with other *P. falciparum var* genes.

## Discussion

**Fig. 5:**
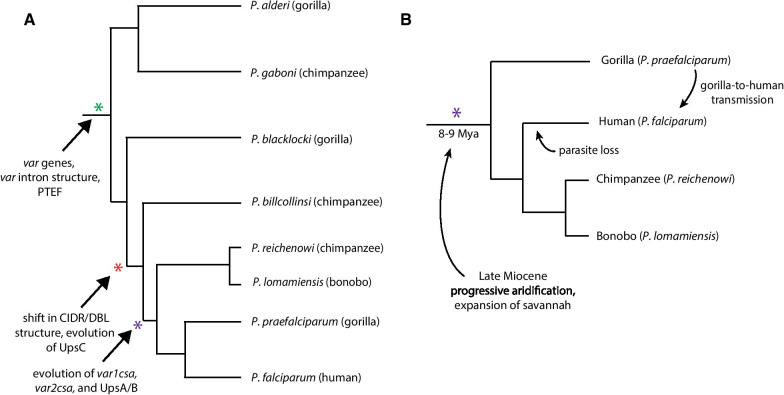
Phylogenetic trees marking key moments in the evolution of the *var* gene family. **A** The evolutionary relationships of the *Laverania* are shown, with each parasite species and its ape host denoted. *var* genes, the regulatory intronic element and the putative regulatory protein PTEF are thought to have originated in the common ancestor of the entire subgenus (shown by green asterisk). A significant shift in the structure of the *var* encoded protein EMP1 and the UpsC upstream regulatory type occurred in the common ancestor of the bottom five species (shown by red asterisk) while the evolution of the conserved genes *var1csa* and *var2csa*, as well as the UpsA and B types, occurred prior to the branch that includes the human parasite *P. falciparum* (shown by purple asterisk). **B** The codivergence model of parasite speciation is displayed showing the branch of the *Laverania* that evolved *var1csa*, *var2csa* and the UpsA/B/C organization. The phylogenetic tree shows the evolution of gorillas, humans, chimpanzees and bonobos from a common ancestor ~ 8–9 million years ago. This model presumes that parasites species diverged along with their hosts, resulting in *P. praefalciparum*, *P. reichenowi* and *P. lomamiensis* infecting gorillas, chimpanzees and bonobos, respectively. Humans are hypothesized to have lost their original parasites, then become reinfected through a recent gorilla-to-human transmission event, resulting in *P. falciparum*. These ape species (and their parasites) initially diverged during the late Miocene, a period of major climatic change in Africa marked by aridification and a regional shift from rainforests to grasslands and savannah

Both the evolution of the UpsA and B *var* upstream regulatory domain subtypes and the development of the species-transcendent, conserved genes *var1csa* and *var2csa* arose in the common ancestor of *P. praefalciparum*, *P. falciparum* and *P. reichenowi* as well as *P. lomamiensis* [65], a parasite of bonobos with insufficient genome sequence data to be included in the analysis described here (Fig. 5A). Our analysis suggests that a strong selective pressure on parasites at this particular moment in the evolutionary history of the *Laverania* led to the evolution of *var1csa*, *var2csa* and the UpsA/B/C organization of the *var* gene family. Further, this selection pressure appears to have been sufficiently powerful and sustained to maintain these changes as the subsequent parasite species diverged, despite the hyper-recombinogenic nature of the *var* gene family and the plasticity of the subtelomeric regions of the genome, two characteristics that would be predicted to rapidly disrupt this organization in the absence of strong selection. Thus, the selection pressure that led to this change in *var* gene organization likely represents an exceptionally strong influence that shaped the genome of the most virulent of the human malaria parasite species.

The functional significance of the development of the *var1csa*/*var2csa*/UspA/B/C organization of the *var* gene family is unknown but is likely to involve how the family is transcriptionally regulated. Mathematical modelling has previously proposed that coordination of *var* gene transcriptional switching might be achieved through central organizing *var* genes that serve as nodes within a switching network [66], and additional work has implicated *var2csa* in this function [19, 20]. If this model holds, *var1csa* might similarly contribute to coordinating *var* gene switching patterns, thereby providing an explanation for its universal conservation despite a timing of transcription inconsistent with the production of EMP1 and its annotation as a pseudogene. The divergence of *var* upstream transcriptional regulatory domains into UpsA/B/C/D/E types could provide additional structure to the switching network, thereby organizing the family into a hierarchy of genes with different switching rates. This would influence the probability of specific genes being activated at different time points of an infection or in the presence of different degrees of host immunity, as has been observed [59, 60, 67], and would contribute to a coordinated order of gene activation. More sophisticated coordination of *var* gene switching has been proposed as a way for parasite populations that can number in the billions of individuals to limit *var* gene expression to a single or very small number of *var* genes at any given time [66]. In contrast, uncoordinated, random *var* gene switching by individual parasites within such large circulating populations would result in much more rapid immune exposure of the entire *var* repertoire. Therefore, we hypothesize that the evolution of *var1csa*, *var2csa* and the UpsA/B/C/D/E organization of the *var* gene family enabled parasite populations to maintain more lengthy infections, although why this trait would have been under such strong selection pressure specifically in the common ancestor of this clade of the *Laverania* is not immediately apparent.

To better understand what might have given rise to the evolution of a more coordinated mechanism of *var* gene transcriptional regulation, we considered the current models for the speciation of the *Laverania*. Using estimates of mutation rates and Bayesian whole-genome estimates, Otto and colleagues dated the origins of the *Laverania* to 0.7–1.2 million years ago, and the common ancestor of *P. reichenowi*/*P. praefalciparum*/*P. falciparum* to 140–230 thousand years ago [12]. Others have also dated speciation events within the *Laverania* to timepoints in the relatively recent past [68]. In contrast, Hahn and colleagues proposed a much older origin of this parasite clade. Their analysis indicates that the species within this branch of the *Laverania* arose through codivergence with their ape hosts, beginning with the ancestors of gorillas and chimpanzees approximately 8–9 million years ago (Fig. 5B) [65, 69]. This model suggests that humans lost their initial *Laverania* parasites after diverging from chimpanzees, and subsequently acquired *P. falciparum* through a relatively recent gorilla-to-human transmission event [70]. The latter model is particularly intriguing since it places the common ancestor of this parasite clade within the late Miocene period, a time of substantial climatic and environmental change on the African continent that is known to have contributed to ape speciation [71]. Specifically, the continent experienced progressive cooling and aridification, resulting in the contraction of continent-wide rainforests and a drastic expansion of grasslands and savannah (reviewed in [72]). This change in environmental conditions would have created large geographical ranges with seasonal transmission, including lengthy time periods in which malaria transmission is greatly reduced or eliminated, for example dry seasons when the mosquito vector can be absent for months. In order to sustain transmission under such conditions, parasites would have been required to extend individual infections to span such extended time intervals when transmission is absent. This is precisely what more coordinated *var* gene transcriptional switching patterns have been proposed to do [66], and provides a compelling hypothesis for the selection pressure that underlies the origin and maintenance of *var1csa*/*var2csa*/UpsA/B/C/D/E organization of the gene family. While the current geographical distribution of both host and parasite species have been documented [65, 69], the selective pressures contributing to these distributions have not been identified, and may or may not reflect the selective pressures in place when the *var1csa*/*var2csa*/UpsA/B/C/D/E organization evolved. Similarly, the chronicity of infections with species other than *P. falciparum* have not been rigorously investigated. Thus, this hypothesis remains speculative.

In conclusion, the strategy of avoiding splenic clearance through cytoadherence, a phenomenon that lies at the heart of the exceptional virulence of *P. falciparum* in humans, appears to have evolved in the common ancestor of the entire *Laverania* subgenus. Along with the *var* gene family and the cytoadhesive protein EMP1, the basic elements for transcriptional regulation of this family, including the proteins implicated in the epigenetic regulation of transcriptional activation, silencing and mutually exclusive expression, were present in the common ancestor of all *Laverania*. This also includes the regulatory element found in *var* introns and its associated noncoding RNAs. Thus, strong cytoadhesion and tightly regulated antigenic variation based on mutually exclusive *var* gene expression are universal characteristics of these parasites and represent an ancient, conserved evolutionary adaptation of the *Laverania*. However, not all aspects of *var* gene regulation and cytoadhesion through EMP1 expression have remained unchanged and at least two significant events have helped shape the evolution of the most virulent of the malaria species that infect humans (Fig. 5A). In addition to changes in *var* transcriptional regulation, Otto and colleagues previously documented a significant alteration in EMP1 structure and speculated that this could indicate a change in the host cell surface receptors used for cytoadherence [12]. How these changes in cytoadhesive properties affected interactions with their hosts is not known, however they have been incorporated into a highly successful human pathogen that continues to cause substantial morbidity and mortality throughout the developing world. Understanding the key evolutionary events that shaped this subgenus of parasites provides valuable insights into the pathogenesis and persistence of malaria caused by *P. falciparum*.

### Methods

### *var* Intron structure analysis

Analysis of *var* intron base pair content was performed as described by Calderwood et al. [41]. Briefly, 10 *var* genes were randomly selected for each species in the *Laverania* clade from the genome sequence database at EupathDB.org. Each gene was uploaded as an individual file into the sequence analysis program SnapGene (www.snapgene.com) and the intron was manually divided into each region based on base content as described by Calderwood et al. [41]. The percentage of each base on the positive strand was calculated per region, averaged, and graphed using Prism (www.graphpad.com).

### Presence of regulatory proteins, *var1csa*, and *var2csa* in the *Laverania* Clade

The histone methyltransferases SET2 [21, 22] and SET10 [23], the demethylase JmJC1 [22, 24], the histone deacetylases SIR2A and SIR2B [25–27], the translation factor PTEF [28] and the RECQ helicases RQ1 and WRN [29, 30] were chosen based on their proposed roles in the regulation of *var* gene expression. The amino acid sequences and the genomic position of each gene from the 3D7 reference genome of *P. falciparum* were obtained from the EupathDb database (www.plasmodb.org) and analysed using the Basic Local Alignment Search Tool (BLAST; ncbi.nlm.gov). The top hit corresponded to the orthologous gene in each species, and the syntenic chromosomal location for each gene was verified. With the exception of PTEF in *P. blacklocki* (incomplete sequence assembly), every gene resided in the syntenic location based on the 3D7 genome. For *var1csa* and *var2csa*, the same procedure was performed.

### Upstream *var* regulatory region analysis

*Collecting sequences*: Analysis of *var* upstream sequences was performed as described by Kraemer and Smith [57]. The *var* genes from each species were compiled by downloading 1.5 kb upstream of each “PfEMP1” or “EMP1” labeled gene on PlasmoDB. The number of compiled sequences were compared with the number of genes reported by Otto et al. [12], and in all cases, the initial number of genes we collected exceeded the number previously reported. Each gene was reevaluated based on the following criteria: Genes with upstream sequences below 500 bp or that had a truncated exon 1 were removed. Similarly, genes with greater than 2 introns or that contained ambiguous nucleotides labeled as “N” in the upstream sequences were discarded unless the ambiguous nucleotides could be deleted from the 5’ end of the sequence without going below 500 bp. Genes on unassembled chromosomes were noted. A full description of the sequences from each species that were included in the analyses is provided in Additional file 2, and a description of the number of genes found for each species analyzed in this study compared to that described by Otto et al. [12] is provided in Additional file 1, Table 1.

*Determining Chromosomal Location and Orientation:* Chromosomal location and orientation for each *var* gene was determined as described by Lavstsen et al. [45] and Kraemer et al. [57]. Subtelomeric regions included sequences within 100 kb of either chromosome end while sequences between the two subtelomeric regions were annotated as being within a chromosomal internal region. The orientation of subtelomeric genes was determined based on direction of transcription, either toward or away from the adjacent telomere. For genes located on unassembled chromosomes, chromosomal location could sometimes be inferred by locating the nearest conserved, single copy gene, then determining the chromosome position of its orthologous gene within the 3D7 reference genome sequence.

*Constructing maximum-likelihood phylogenetic trees:* The collected sequences for the *var* upstream regions from each species were aligned via Clustal Omega (https://www.ebi.ac.uk/Tools/msa/clustalo/) [73] and the resulting FASTA sequences were analyzed using MEGA-X [64], a molecular evolutionary genetics analysis tool (https://www.megasoftware.net/). The ML tree was generated with the Jukes-Cantor Model (63) and 1000 bootstrap replicates. The ML Heuristic Method used was Nearest-Neighbor-Interchange (NNI) and BioNJ.

## Supplementary Information

**Additional file 1**: Maximum-likelihood phylogenetic trees of the 0.5–1.5 kb upstream regulatory regions of *var* genes from all seven *Laverania* species and Collected Ups sequences from all species compared with the number of genes reported by Otto et al., 2018.

**Additional file 2**: Description of individual sequences analyzed. This file provides a description of each sequence analyzed, organized according to species. Specific characteristics for each sequence are provided.

## Data Availability

The datasets analysed during the current study are available in the Eupathdb database (Plasmodb.org). In addition to the datasets available through the Eupathdb database, additional genome sequence data were obtained through the databases originally described by Otto et al., *Nature Microbiology*, 2018. These sequences were submitted to EBI, project ID PRJEB13584 (secondary study accession: ERP015144). These can also be accessed via ftp://ftp.sanger.ac.uk/pub/project/pathogens/Plasmodium/Laverania/.
