# Supplementary material for: Evolution of transcriptional control of antigenic variation and virulence in human and ape malaria parasites
Source: BMC Ecol Evol. 2021 Jul 8;21:139. doi: 10.1186/s12862-021-01872-z (PMC8265125; doi:10.1186/s12862-021-01872-z)
Supplement: Supplementary file 1 — Additional file 1: Maximum-likelihood phylogenetic trees of the 0.5–1.5 kb upstream regulatory regions of var genes from all seven Laverania species and Collected Ups sequences from all species compared with the number of genes reported by Otto et al., 2018. [file 12862_2021_1872_MOESM1_ESM.docx]

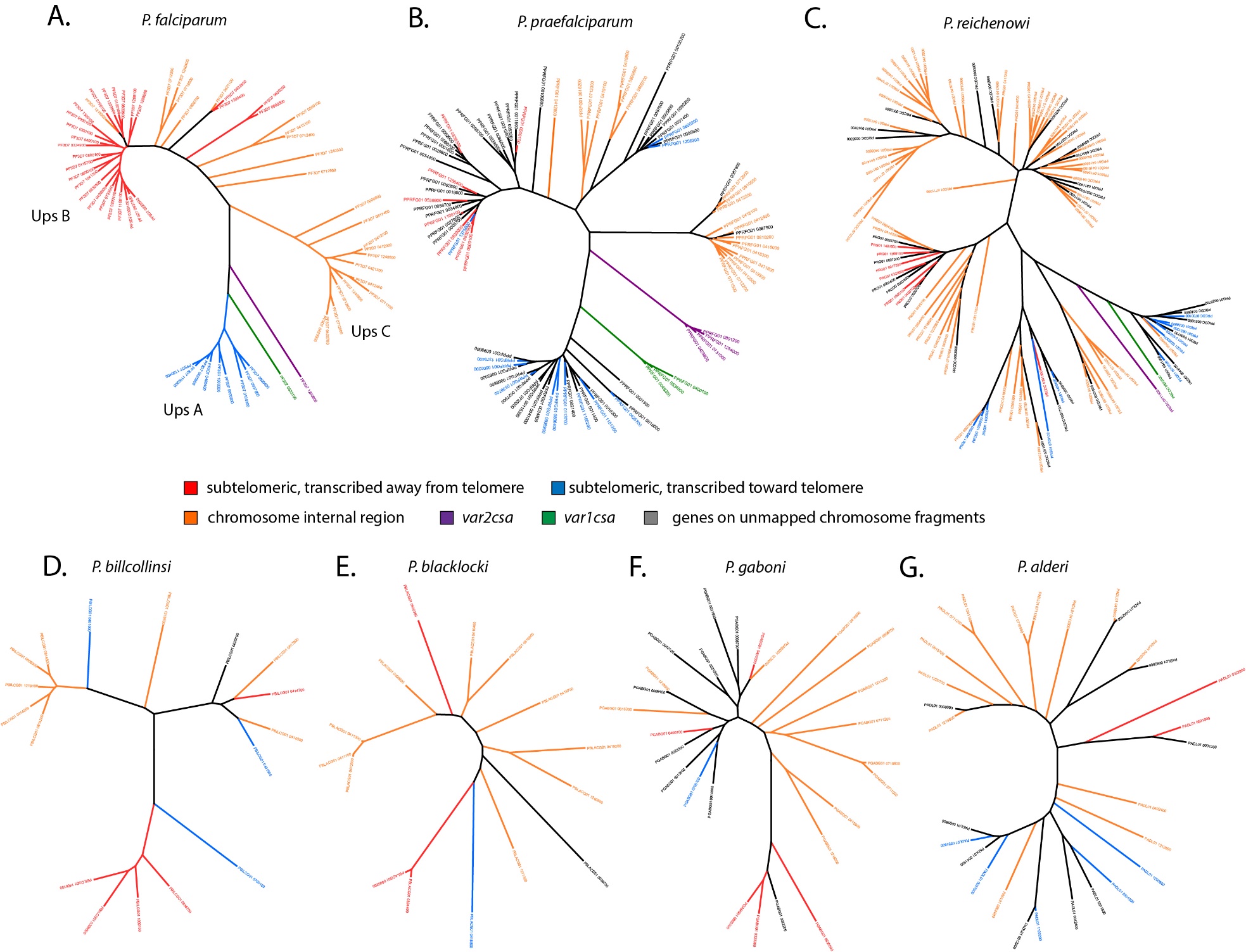


**Figure 1**: Maximum-likelihood phylogenetic trees of the 0.5-1.5 kb upstream regulatory regions of *var* genes from all seven *Laverania* species. The annotation number for each gene (from Plasmodb.org) is shown and the chromosomal position and orientation are denoted by colour (orange for internal genes, red for subtelomeric genes transcribed away from the telomere and blue for subtelomeric genes transcribed toward the telomere). The sequences for P. reichenowi include genes obtained from both the CDC and G01 strains. The conserved genes *var1csa* and *var2csa* are marked in green and purple, respectively. Black text denotes genes on unassigned chromosome fragments.

|  | Otto et. al | Number of Ups sequences analyzed | Genes on unassigned chromosomes |
| --- | --- | --- | --- |
| *P. falciparum* | 67 | 62 | 0 |
| *P. praefalciparum* | 112 | 86 | 39 |
| *P. reichenowi G01/CDC* | 92 | 110 | 30 |
| *P. billcolinsi* | 35 | 17 | 1 |
| *P.blacklocki* | 43 | 15 | 1 |
| *P. gaboni* | 61 | 26 | 9 |
| *P. adleri* | 58 | 29 | 10 |

**Table 1.** Collected Ups sequences from all species compared with the number of genes reported by Otto et al., 2018 (blue). The number of Ups sequences analyzed (yellow) shows the number of sequences that met the criteria required for analysis (see methods section of main text). The unassigned genes (yellow) are included in the total number of Ups sequences analyzed and show the number of genes that reside on DNA fragments that have not be assigned to specific chromosomes.
